# A Comparison of Inflammatory and Oxidative Stress Markers in Adipose Tissue from Weight-Matched Obese Male and Female Mice

**DOI:** 10.1155/2012/859395

**Published:** 2012-06-14

**Authors:** Karen J. Nickelson, Kelly L. Stromsdorfer, R. Taylor Pickering, Tzu-Wen Liu, Laura C. Ortinau, Aileen F. Keating, James W. Perfield

**Affiliations:** ^1^Department of Nutrition and Exercise Physiology, University of Missouri-Columbia, 256 William Stringer Wing, Columbia, MO 65211, USA; ^2^Department of Animal Science, Iowa State University, Ames, IA 50011, USA; ^3^Department of Food Science, University of Missouri-Columbia, 256 William Stringer Wing, Columbia, MO 65211, USA

## Abstract

Expansion of intra-abdominal adipose tissue and the accompanying inflammatory response has been put forward as a unifying link between obesity and the development of chronic diseases. However, an apparent sexual dimorphism exists between obesity and chronic disease risk due to differences in the distribution and abundance of adipose tissue. A range of experimental protocols have been employed to demonstrate the role of estrogen in regulating health benefits; however, most studies are confounded by significant differences in body weight and adiposity. Therefore, the purpose of this study was to compare weight-matched obese male and female mice to determine if the sex-dependent health benefits remain when body weight is similar. The development of obesity in female mice receiving a high-fat diet was delayed; however, subsequent comparisons of weight-matched obese mice revealed greater adiposity in obese female mice. Despite excess adiposity and enlarged adipocyte size, obese females remained more glucose tolerant than weight-matched male mice, and this benefit was associated with increased expression of adiponectin and reductions in immune cell infiltration and oxidative stress in adipose tissue. Therefore, the protective benefits of estrogen persist in the obese state and appear to improve the metabolic phenotype of adipose tissue and the individual.

## 1. Introduction

Obesity is widely regarded as an independent risk factor for a range of chronic diseases including type 2 diabetes and cardiovascular disease [1, 2]. Low-grade systemic inflammation has been put forward as a unifying link between obesity and the onset of these obesity-associated diseases [3–5]. Expansion of intra-abdominal adipose tissue is associated with increased infiltration and activation of immune cells, and these events are a significant contributor to the systemic inflammation that occurs with obesity [6, 7]. While an exact explanation for the accumulation of immune cells in adipose tissue is unknown, one potential contributing factor is elevated oxidative stress [8, 9]. Therefore, decreasing intra-abdominal obesity and/or reducing adipose tissue oxidative stress and inflammation will positively influence chronic disease risk. 

Clear sex-based differences exist in adipose tissue distribution, inflammation, and ultimately the probability of developing a chronic disease [10–12]. Specifically, females tend to have a higher body fat content with the fat localized subcutaneously while males have less total body fat and their adipose tissue predominates in the visceral region. Furthermore, animal studies have demonstrated that diet-induced obesity and insulin resistance occur much more rapidly in male rodents as compared to females [13–15]. Estrogen is a major factor involved in this sexual dimorphism as it promotes subcutaneous fat accumulation, has anti-inflammatory properties, and is a strong regulator of appetite and energy expenditure [10, 12, 16, 17]. To help elucidate the effects of estrogen on obesity, adipose tissue distribution, inflammation, and insulin resistance studies have utilized models of ovariectomy with or without repletion of estrogen and/or compared male and female mice provided a high-fat diet [13–15, 18]. While the outcome measures of these studies varied, they all clearly demonstrate a beneficial effect of estrogen. However, these studies were also confounded by body weight differences as intact females or animals receiving estrogen were typically smaller and had smaller adipose tissue depots.

Therefore, the purpose of the current study was to compare weight-matched obese male and female mice to determine if the sex-dependent improvements in metabolic health occur independent of differences in body weight. Following chronic exposure to a high-fat diet, a glucose tolerance test was performed and differences in markers for inflammatory and oxidative stress were assessed in adipose tissue. Our data demonstrate that glucose tolerance remains improved in obese female mice independent of a difference in body weight. Furthermore, despite increases in total adiposity and gonadal adipocyte size, the obese female mice displayed lower expression of markers for immune cells and oxidative stress which are consistent with an improved metabolic phenotype.

## 2. Methods

### 2.1. Animals and Animal Care

The University of Missouri Animal Care and Use committee approved all procedures involving mice. Animals were maintained at a controlled temperature (22°C) and a 12-hour light: 12-hour dark cycle. Six-to eight-week old male and female C57BL/6 mice were individually housed and fed either a chow (Purina 5001; 4.5 g/100 g fat) or high-fat diet (HFD; Research Diets D12492; 35 g/100 g fat) for the duration of the experiment. Body weight was measured weekly and mice were kept on treatment until the average body weight of the HFD group was 45 g. At this point, glucose tolerance testing and tissue collection were performed on the HFD group and their age-matched chow-fed counterparts.

### 2.2. Glucose Tolerance Testing

Once the HFD-fed group reached a body weight of 45 g; a glucose tolerance test was performed in both HFD and chow-fed animals. Following an overnight fast, a baseline blood sample was taken from the tail vein at time 0. Then an intraperitoneal injection of glucose (1 g/kg BW) was administered and blood glucose concentrations were determined using a handheld glucometer at 30, 60, 90, and 120 minutes postinjection. Glucose area under the curve (AUC) calculations were performed using GraphPad Prism 4 software.

### 2.3. Tissue Collection

One week after the glucose tolerance tests were performed, animals were fasted 10–12 hours and blood glucose was measured via a tail nick. Animals were then euthanized by CO_2_ asphyxiation followed by exsanguination via cardiac puncture. Plasma was separated by centrifugation, aliquoted, and frozen for future analysis. Gonadal and subcutaneous adipose tissues were excised, weighed, and snap frozen for gene expression analysis or fixed for histological analysis.

### 2.4. Histological Analysis of Adipose Tissue

A portion of the gonadal adipose tissue was fixed in 4% paraformaldehyde, embedded in paraffin, sectioned, and stained with hematoxylin and eosin (H&E). Digital images were acquired with an Olympus BX51 light microscope using an Olympus DP70 camera. Dead adipocytes were quantified by identification of crown-like structures (CLSs) within histologic sections of adipose tissue. The percentage of CLS present in gonadal adipose tissue was calculated and used for comparison among experimental groups. Adipocyte volume was calculated using the cross-sectional area obtained from perimeter tracings using Image J software (Sun Microsystems, Santa Clara, CA, USA).

### 2.5. Plasma Analysis

An estradiol EIA kit (Cayman Chemical Company) was used to determine fasting (overnight) plasma estradiol concentrations of female mice.

### 2.6. Real-Time Quantitative PCR

Total mRNA was extracted from adipose using RNeasy lipid tissue kits with on-column DNase digestion (Qiagen). Purity and concentration were determined with a Nanodrop 1000 spectrophotometer (Thermo Scientific). 1 *μ*g of RNA was used to synthesize cDNA with a reverse transcriptase polymerase chain reaction kit (Applied Biosystems) and diluted to 10 ng/*μ*L. Expression of mRNA was determined using SYBR green qRT-PCR on an Applied Biosystems StepOne Plus RT-PCR system. Fold difference for gene expression was calculated using 2^−ΔΔCT^ using the endogenous control gene RPS-3.

### 2.7. Statistical Analysis

Treatment differences were analyzed by one-way analysis of variance (ANOVA) with main effect significance set at *P* < 0.05. Significant main effects were followed by a Tukey's multiple comparison test. Values are reported as mean ± standard error.

## 3. Results

### 3.1. Onset of Obesity Is Delayed in Female Mice Receiving High-Fat Diet

Male and female C57BL/6 J mice received either a low-fat chow diet or a HFD. The HFD induced a more rapid body weight gain in male mice which achieved the target body weight of 45 g in 21 weeks, while female mice required an additional 17 weeks of HFD to reach the same body weight (Table 1). This experimental design ensured that mice would be studied at a body weight associated with established obesity and insulin resistance [6] and that body weight would not be a confounding variable. However, due to the delayed body weight gain in female HFD mice age did emerge as an unaccounted for variable. Interestingly, despite having similar body weights, the adiposity of the female HFD mice was greater than the male HFD group as both gonadal and subcutaneous adipose tissue masses were significantly increased (Table 1). Fasting blood glucose concentrations were elevated by obesity in the male mice but not the female mice. Obesity also did not alter plasma estradiol concentrations in the female mice (Table 1).

### 3.2. Obese Female Mice Have Improved Glucose Tolerance as Compared to Weight-Matched Obese Male Mice

**Figure 1:**
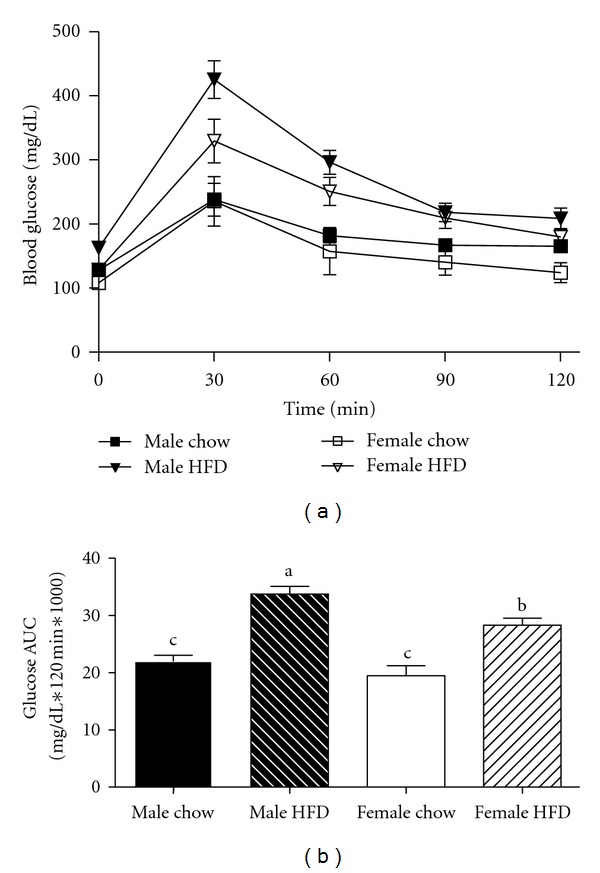
Obese female mice (female HFD) have improved glucose tolerance when compared to weight-matched obese male mice (male HFD). Male and female C57BL/6 mice were fed either a standard rodent chow or a high-fat diet (HFD) from 6 weeks of age until the HFD-fed group achieved a body weight of 45 g. At that time, a glucose tolerance test was performed in both chow- and HFD-fed males (21 weeks old) or females (38 weeks old) and blood glucose change over time plotted (a). Corresponding blood glucose area under the curve (AUC) was calculated (b), data are reported as mean ± SE and means with different superscripts differ by *P* < 0.05. *n* = 5–8 per group.

When the average body weight of HFD-fed male and female mice reached 45 g, an intraperitoneal glucose tolerance test was performed on that experimental group and their age-matched chow-fed counterparts. Glucose area under the curve calculations revealed better glucose tolerance in the female HFD group as compared to the male HFD mice (Figure 1). This improvement is especially noteworthy since there was no difference in body weight between these two groups and also, the HFD females had greater total fat mass (Table 1). Further, despite the age difference, glucose tolerance was similar between male chow and female chow mice (Figure 1).

### 3.3. Immune Cell Infiltration and Oxidative Stress Markers Are Reduced in the Adipose Tissue of Obese Female Mice

**Figure 2:**
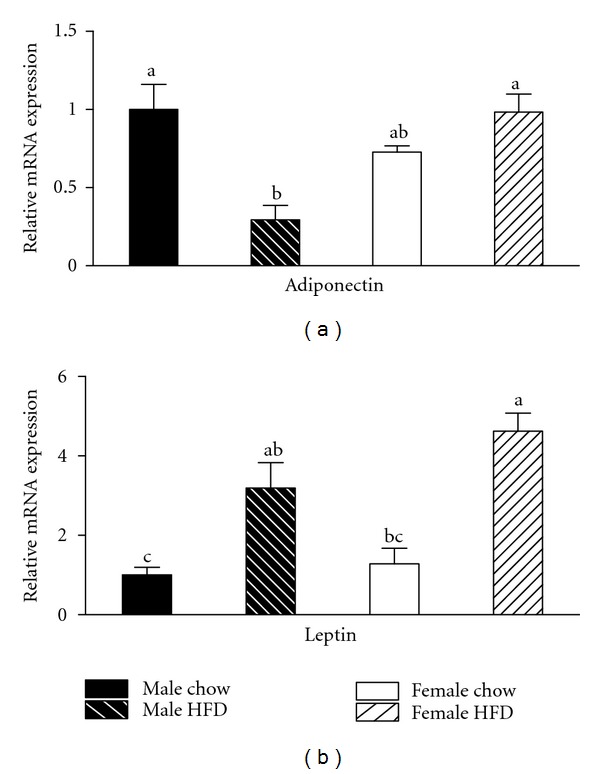
Adiponectin mRNA expression in gonadal adipose tissue is reduced by obesity in male mice but not weight-matched obese female mice. Male and female C57BL/6 mice were fed either a standard rodent chow or a high-fat diet (HFD) from 6 weeks of age until the HFD-fed group achieved a body weight of 45 g. At that time, both chow- and HFD-fed males (21 weeks old) or females (38 weeks old) were sacrificed and qRT PCR performed on gonadal adipose tissue. Relative mRNA expression of adiponectin was reduced by obesity in male mice, while obesity had no effect on adiponectin expression in female mice (a). Consistent with an obese phenotype, mRNA expression of leptin was elevated in both male HFD and female HFD mice (b). Data are reported as mean ± SE; *n* = 5–8 per group; means with different superscripts differ by *P* < 0.05.

**Figure 3:**
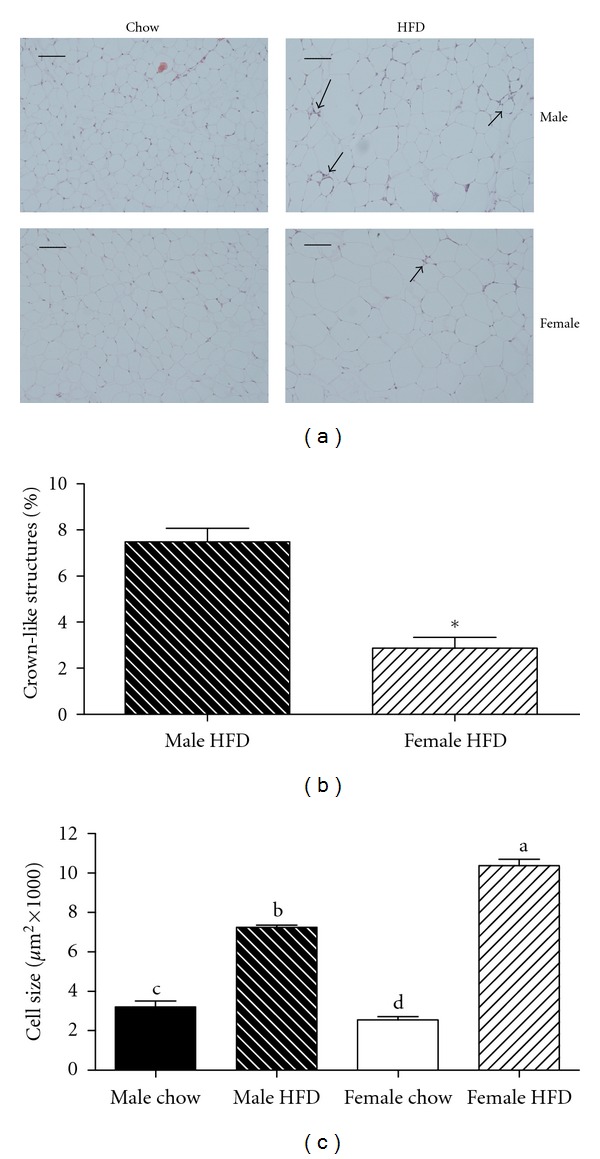
Obese female mice have larger adipocytes and reduced prevalence of crown-like structures in gonadal adipose tissue when compared to weight-matched obese male mice. Male and female C57BL/6 mice were fed either a standard rodent chow or a high-fat diet (HFD) from 6 weeks of age until the HFD-fed group achieved a body weight of 45 g. At that time, both chow- and HFD-fed males (21 weeks old) or females (38 weeks old) were sacrificed and histological analysis was performed on gonadal adipose tissue. Representative H&E stains of gonadal adipose tissue from each of the four treatment groups are presented in panel (a). Sections were used to quantify the presence of crown-like structure (b) and to calculate average adipocyte area (c). Data are reported as mean ± SE; *n* = 5–8 per group; means with different superscripts differ by *P* < 0.05; *P < 0.05; bar = 100 *μ*M.

In order to better understand the observed glucose tolerance differences, we characterized markers of inflammation and oxidative stress in gonadal adipose tissue. In the current study, gonadal adipose tissue mass was greater in female HFD mice as compared to the male HFD group (Table 1). Consistent with an increased mass of adipose tissue, relative mRNA expression of leptin in gonadal adipose tissue was elevated in both male HFD and female HFD groups with no difference between genders observed (Figure 2). Interestingly, gonadal adipose tissue mRNA expression of adiponectin was reduced in male HFD mice but remained unchanged in female HFD mice (Figure 2). Adiponectin expression is often correlated with smaller adipocyte size. Therefore, gonadal adipocyte size was quantified, and surprisingly female HFD mice had a larger average adipocyte size as compared to male HFD mice (Figure 3). This apparent disconnect between adipocyte size and adiponectin expression may help explain the improvements in glucose tolerance.

**Figure 4:**
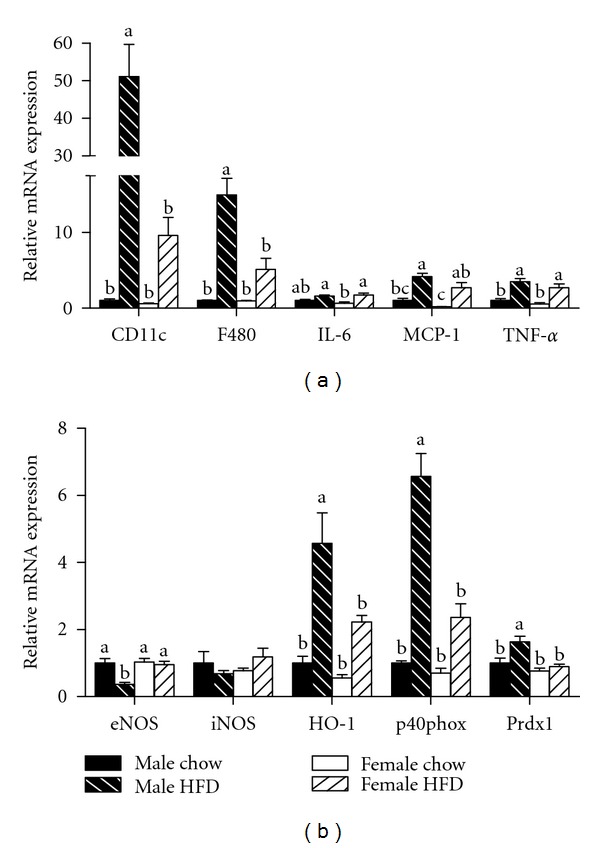
Relative mRNA expression of markers for immune cell infiltration and oxidative stress is decreased in gonadal adipose tissue isolated from obese female mice as compared to obese male mice. Male and female C57BL/6 mice were fed either a standard rodent chow or a high-fat diet (HFD) from 6 weeks of age until the HFD-fed group achieved a body weight of 45 g. At that time, both chow- and HFD-fed males (21 weeks old) or females (38 weeks old) were sacrificed and qRT PCR was performed on gonadal adipose tissue. Relative mRNA expression of markers for immune cell infiltration and inflammation (a) as well as oxidative stress (b) was determined. Data are reported as mean ± SE; *n* = 5–8 per group; means with different superscripts differ by *P* < 0.05. IL-6: interleukin-6; MCP-1: monocyte chemoattractant protein-1; TNF-*α*: tumor necrosis factor-alpha; eNOS: endothelial nitric oxide synthase; iNOS: inducible nitric oxide synthase; HO-1: heme oxygenase-1; p40phox: NADPH subunit p40phox; Prdx1: peroxiredoxin-1.

Increases in adipocyte size also are correlated with an increased presence of immune cells in adipose tissue [6, 19]. Crown-like structures (CLSs) are clusters of proinflammatory immune cells that localize to dead adipocytes within adipose tissue [20]. Consistent with previous reports [6, 21], gonadal adipose tissue from the male HFD group contained elevated numbers of CLS (Figure 3). Interestingly, the presence of CLS in the gonadal adipose tissue of female HFD mice was reduced by greater than 50% when compared to the male HFD group (Figure 3). In support of this observed reduction, mRNA expression of the macrophage markers F480 and CD11c were also decreased in female HFD compared to male HFD mice (Figure 4). However, there was no difference in the relative expression of the inflammatory cytokines IL-6 and TNF-*α* or the chemokine MCP-1 (Figure 4). Obesity caused a reduction in the relative mRNA expression of eNOS in male HFD gonadal adipose tissue while there was no change in the female HFD group (Figure 4). Furthermore, the oxidative stress markers HO-1, p40phox, and prdx1 were increased in male HFD gonadal adipose tissue, and this increase was attenuated in gonadal adipose tissue from female HFD mice (Figure 4). Importantly, when gonadal adipose tissue from male chow and female chow mice was compared, adipocyte size was smaller in female mice, but there were no differences in any of the other variables that were measured (Figures 2, 3, and 4). Overall, adipocyte size and adipose tissue masses were greater in weight-matched female HFD mice as compared to male HFD mice. However, female HFD mice had reduced immune cell infiltration and oxidative stress, and this may be due, in part, to increased adiponectin expression.

**Figure 5:**
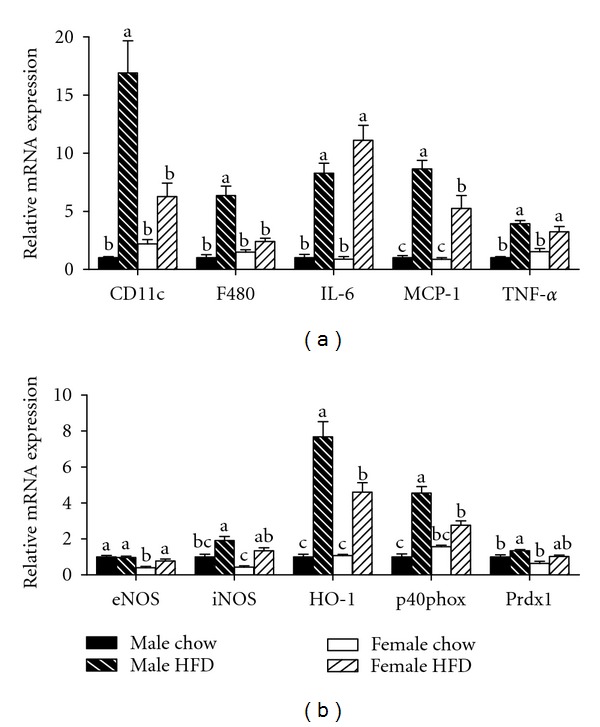
Relative mRNA expression of markers for immune cell infiltration and oxidative stress is altered in subcutaneous adipose tissue isolated from obese female mice as compared to obese male mice. Male and female C57BL/6 mice were fed either a standard rodent chow or a high-fat diet (HFD) from 6 weeks of age until the HFD-fed group achieved a body weight of 45 g. At that time, both chow- and HFD-fed males (21 weeks old) or females (38 weeks old) were sacrificed and qRT PCR was performed on subcutaneous adipose tissue. Relative mRNA expression of markers for immune cell infiltration and inflammation (a) as well as oxidative stress (b) was determined. Data are reported as mean ± SE; *n* = 5–8 per group; means with different superscripts differ by *P* < 0.05. IL-6: interleukin-6; MCP-1: monocyte chemoattractant protein-1; TNF-*α*: tumor necrosis factor-alpha; eNOS: endothelial nitric oxide synthase; iNOS: inducible nitric oxide synthase; HO-1: heme oxygenase-1; p40phox: NADPH subunit p40phox; Prdx1: peroxiredoxin-1.

Subcutaneous adipose tissue has been reported to be metabolically different than gonadal adipose tissue [22] and therefore the gene expression profile of this tissue was also investigated. Subcutaneous adipose tissue mRNA expression of CD11c and F480 was increased in male HFD mice as compared to the female HFD group (Figure 5). Obesity also caused an increase in MCP-1 expression in this tissue; however, the increase was greater in the male HFD animals as compared to the female HFD mice. The expression of the inflammatory cytokines IL-6 and TNF-*α* was increased in both male HFD and female HFD mice (Figure 5). Similar to the results reported for gonadal adipose tissue, mRNA expression of oxidative stress markers in subcutaneous adipose tissue was elevated in male HFD mice as compared to the female HFD group (Figure 5). In contrast to the gonadal adipose tissue, we did not observe any differences in adiponectin expression in subcutaneous adipose tissue (data not shown). No appreciable differences in subcutaneous mRNA expression for any of the genes investigated were observed between male chow and female chow groups (Figure 5).

## 4. Discussion

While numerous studies have demonstrated the benefits of estrogen in obesity prevention and chronic disease risk management [13–17, 23], our study is novel in that weight-matched obese male and female mice were evaluated to determine if endogenous estrogen provides health benefits in the obese state that are independent of body weight differences. As observed from the circulating estradiol levels, these female mice had not entered ovarian senescence. Postmenopausal mice have been shown to display a more severe obese phenotype relative to their cycling, age-matched controls [24], further supporting that estradiol provides protection against HFD-induced obesity and alternations in glucose metabolism. Consistent with previous reports [13–15], we observed that male mice were more susceptible to HFD-induced obesity as compared to the female HFD group. Therefore, we recognize that age is a potential confounding variable that was not accounted for in the current study design as it took 17 weeks longer for the female HFD group to reach the target body weight of 45 g. This target body weight was selected because it has been shown to be a period of established obesity, adipose tissue inflammation, and insulin resistance in male mice [6]. 

Regardless of the difference in age, obese female mice had improved glucose clearance during a glucose tolerance test as compared to weight-matched obese males. This improvement in glucose metabolism was supported by the observation that obesity caused an increase in fasting blood glucose concentrations in males but not females when compared to their chow-fed littermates. These data demonstrate that chronic exposure to a HFD can induce an obese phenotype in female mice; however, the development of insulin resistance in these animals is not as severe as that observed in weight-matched obese male mice. In addition, advanced age is associated with the development of insulin resistance [25] suggesting the improvement in glucose tolerance may have been greater if the significant difference in age did not exist between the two groups. This difference in age may explain why glucose tolerance was similar between the male chow and female chow-groups while others have reported differences in glucose tolerance testing between chow-fed male and female mice [26].

Given the strong correlation between metabolic dysfunction in adipose tissue and impaired glucose metabolism; we examined the adipose tissue from the four experimental groups in an attempt to better understand the observed improvement in glucose tolerance in obese females. The experimental design precluded body weight differences between male HFD and female HFD groups; however, body fat content of the female HFD mice was greater due to increased gonadal and subcutaneous adipose tissue mass. This increase in mass was associated with increased adipocyte size in the gonadal adipose tissue and is contrary to our observation in chow-fed animals and the reports of others studying lean animals [26]. Both increased intra-abdominal fat mass and adipocyte size have been associated with insulin resistance in male mice due to an increased infiltration and activation of proinflammatory immune cells in the adipose tissue [6, 7, 19, 27, 28]. Here we observe an apparent disconnect where female mice have increased adipose tissue mass and larger adipocytes but glucose tolerance is improved. When immune cell infiltration and activation were assessed, we observed a reduction in the appearance of immune cells and the formation of CLS in female HFD gonadal adipose tissue. This correlated with reductions in mRNA expression of CD11c and F480 although expression of proinflammatory cytokines was not different between male HFD and female HFD groups, suggesting that a factor other than these cytokines was potentially responsible for the improvement in glucose tolerance. 

 We then measured mRNA expression of the adipokines leptin and adiponectin in gonadal adipose tissue. Consistent with previous studies [29], obesity caused an increase in leptin expression in both HFD-fed groups. Interestingly, obesity caused a reduction in adiponectin expression in the male HFD group, but expression was unchanged with obesity in the female HFD group. These data are not surprising as females typically have higher adiponectin levels than males [30, 31] and while this appears to occur independent of estradiol levels, adiponectin is associated with improved insulin sensitivity and suggests a potential mechanism for our observed improvement in the females. However, additional studies investigating circulating concentrations of adiponectin are required to more fully explore this possibility. Endothelial dysfunction and oxidative stress are additional stressors that can influence glucose metabolism and were assessed in adipose tissue. Reduced mRNA expression of eNOS and elevations in HO-1, p40phox, and prdx1 in the adipose tissue of male HFD mice is indicative of oxidative stress within the tissue [32–35]. Alterations in the profile of these genes were not as severe in the female HFD adipose tissue revealing a possible reduction in oxidative stress and another potential mechanism by which estrogen may have provided a beneficial effect. Nominal differences existed between male chow and female chow adipose tissue and therefore it is unlikely that any of our observations in the obese cohorts were influenced by baseline differences.

Overall, our study demonstrates that if provided enough time, chronic exposure to a hypercaloric diet will induce an obese phenotype in female mice that is characterized by excess abdominal adiposity and enlarged adipocytes as compared to weight-matched obese male mice. However, despite being an older animal, female mice maintained partial protection from the detrimental effects of obesity as demonstrated by improved glucose tolerance testing. Furthermore, immune cell infiltration and oxidative stress were reduced in the adipose tissue of obese female mice, and these changes were associated with increased adiponectin expression. It is likely that a combination of these factors is responsible for the observed improvement in glucose tolerance. While obesity did not alter circulating levels of estrogen we recognize that estrogen may not be the only factor influencing the improved phenotype observed in the female HFD group. However, our data are consistent with studies that have more carefully manipulated circulating levels of estrogen. Future studies in weight-matched obese females will be required to extend and verify these initial findings. 

## Figures and Tables

**Table 1:** Characteristics of male and female C57BL/6 mice receiving a standard rodent chow or a high-fat diet (HFD).^1^

	Male chow	Male HFD	Female chow	Female HFD
Body weight (g)	28.8 ± 0.28^b^	45.3 ± 1.0^a^	24.8 ± 0.45^b^	46.5 ± 2.0^a^
Age^2^ (weeks)	21	21	38	38
Gonadal AT^3^ (g)	0.51 ± 0.28^c^	2.25 ± 0.17^b^	0.44 ± 0.05^c^	3.74 ± 0.35^a^
Subcutaneous AT (g)	0.32 ± 0.01^c^	2.29 ± 0.24^b^	0.27 ± 0.02^c^	3.34 ± 0.28^a^
Blood glucose (mg/dL)	99 ± 8^b^	142 ± 11^a^	108 ± 8^b^	116 ± 5^ab^
Plasma estradiol (pg/mL)	—	—	92.5 ± 1.53	92.7 ± 0.35

^1^Data are presented as means ± SE; means with different superscripts differ *P* < 0.05; *n* = 5–8.

^2^Age at which the HFD-fed group reached a body weight of 45 g and metabolic testing and tissue collection occurred.

^3^AT; adipose tissue.
